# Differences of sex development and surgical decisions: focus group interviews with health care professionals in Norway

**DOI:** 10.1080/21642850.2024.2371134

**Published:** 2024-07-06

**Authors:** Line Merete Mediå, Lena Fauske, Solrun Sigurdardottir, Kristin J. Billaud Feragen, Anne Waehre

**Affiliations:** a Women’s and Children’s Division, Centre for Rare Disorders, Oslo University Hospital, Oslo, Norway; b Department of Interdisciplinary Health Sciences, Institute of Health and Society, University of Oslo, Oslo, Norway; c Department of Oncology, Norwegian Radium Hospital, Oslo University Hospital, Oslo, Norway; d Department of Child and Adolescent Psychiatry, Oslo University Hospital and Institute of Clinical Medicine, Oslo, Norway

**Keywords:** Differences of sex development, intersex, surgery, shared decision-making, qualitative

## Abstract

**Background:**

Differences of Sex Development (DSD) are congenital conditions where the chromosomal, gonadal and anatomical sex characteristics do not strictly belong to male or female categories, or that belong to both at the same time. Surgical interventions for individuals with DSD remain controversial, among affected individuals, caregivers, and health-care providers. A lack of evidence in support of, for deferring, or for avoiding surgery complicates the decision-making process. This study explores Norwegian health-care professionals’ (HCPs) perspectives on decision-making in DSD-related surgeries and the dilemmas they are facing in this process.

**Methods:**

Focus group interviews with 14 HCPs integrated into or collaborating with multidisciplinary DSD teams were analyzed using reflexive thematic analysis.

**Results:**

Two overarching dilemmas shed light on the intricate considerations and challenges that HCPs encounter when guiding affected individuals and caregivers through surgical decision-making processes in the context of DSD. The first theme describes how shared decision-making was found to be influenced by fear of stigma and balancing the interplay between concepts of normality, personal experiences and external expectations when navigating the child’s and caregivers’ needs. The second theme illuminated dilemmas due to a lack of evidence-based practice. The core concepts within each theme were the dilemmas health-care professionals face during consultations with caregivers and affected individuals.

**Conclusion:**

HCPs were aware of the controversies with DSD-related surgeries. However, they struggled to reconcile knowledge with parents’ wishes for surgery and faced dilemmas making decisions in the best interests of the child. This study draws attention to the benefits of increased knowledge on the consequences of performing or withholding surgery as well as incorporating tools enabling shared decision-making between HCPs and affected individuals/caregivers.

## HIGHLIGHTS

- DSD-related surgeries are controversial and subject to debate.
- Health-care professionals grapple with dilemmas during shared decision-making, as revealed in focus group interviews.
- Health-care professionals were concerned that parents’ and affected individuals’ fear of stigmatization would influence the shared decision-making process. Lack of evidence-based knowledge on practice, left health care professionals without clear guidelines on how to navigate decision-making.

## Abbreviations and clarification of concepts

DSD: Differences of sex development
FGIs: Focus group interviews
FGs: Focus groups
Hypospadias: affects the development of the penis in males and can present as mild (where the opening of the urethra is close to the tip or on the underside along the shaft) to more severe (where the opening is located closer to the scrotum and the penis may be underdeveloped) (Kumar & Cherian, 2022)
MDT: Multidisciplinary team
HCPs: Health-care professionals
Swyer syndrome: refers to females who have a 46, XY karyotype (typical for individuals identifying as males). Females with Swyer are born with a uterus, vagina, and fallopian tubes, but the gonads are dysgenic and produce no hormones. Early prophylactic removal of the dysgenic gonads is recommended due to the risk of developing gonadal malignancy. Hormonal therapy is required (Michala & Creighton, 2010)

## Introduction

Differences of sex development (DSD) represent a heterogeneous group of congenital conditions that lead to a diverse development of the genitals, hormones, or chromosomes (Cools et al., 2018). DSD necessitate individuals, their caregivers, and health-care professionals (HCPs) making decisions about medical treatment, including hormonal treatment, and, for some, different types of surgeries. The conditions categorized as DSD are rare and vary in terms of the severity, degree of complications, psychological impacts, and treatment needs and possibilities (Hughes et al., 2005). Moreover, DSD may be associated with stigma and controversy (Hegarty & Smith, 2023; Lampalzer et al., 2020), which may adversely affect individuals’ physical and psychosocial health, indicating the need for individualized care and long-term follow-up (Lee et al., 2016). As indicated by clinical guidelines and consensus statements (Coleman et al., 2022; Cools et al., 2018; Hughes et al., 2005), medical treatment, surgical management and follow-up care of people with DSD should be provided by multidisciplinary teams (MDTs).

DSD-related surgeries are procedures performed on the external genitalia (e.g. genital reconstructive surgery, clitoroplasty when female external genitalia are considered larger than expected, which is often described as virilized) or internal reproductive structures (gonads) (Gardner & Sandberg, 2018). Historically, DSD was established as ‘a treatable condition of the genitals, one that needs to be resolved within eighteen months of age’ (Kessler, 1990, p. 6). The treatment refereed to a surgically correction of the child’s ambiguous sex to fit the gender of rearing and to enable bonding between parent and child. This belief was questioned by Kessler, a social scientist, pointing to the fact that the evidence for surgically correcting genitals ‘is based on only a handful of repeatedly cited cases’ proposed by the psychologist John Money (Kessler, 1990, p. 7). Kessler’s view on surgery was supported by patient advocacy and medical ethics (Hegarty et al., 2021). DSD-related surgeries, and surgical interventions on female external genitalia in particular, are controversial and still a subject to debate amongst HCPs and human-rights activists (Lee et al., 2016). In 2006, a consensus statement was published to improve the management of DSD (Hughes et al., 2005). In the statement, there were proposed suggestions for improving DSD care, which required an open communication and a more cautious approach, or even a delay in the decision regarding a change in genital appearance, until the affected person (the child) was able to participate in decision-making. Furthermore, these suggestions are consistent with the clinical guideline for the transgender and gender diverse population updated by the World Professional Association for Transgender Health (Coleman et al., 2022). However, a debate on the best approach is ongoing (Bennecke et al., 2021; Coleman et al., 2022; Flewelling et al., 2022; Lampalzer et al., 2020; Roen, 2019) and existing studies have not documented reduction in numbers of genital surgeries on children with DSD (Creighton & Wood, 2013; Mouriquand et al., 2016). Some studies also indicate that even though HCPs advocate for a non-surgical treatment option, their communication can be unclear, resulting in parents still choosing a surgical option (Liao et al., 2019; Timmermans et al., 2018).

Making decisions concerning DSD-related surgeries can prove problematic for individuals with DSD, their caregivers, and HCPs (Bennecke et al., 2021; Flewelling et al., 2022; Hegarty et al., 2021; Kremen et al., 2022). There are several models for making healthcare decisions in the patient’s best interests. The consensus statement (Lee et al., 2006) opted for a patient centered care model. Timmermans and colleagues (2018, p. 522) problematized how patient-centered DSD care may lead to different outcomes when the ‘relationship between patient and physician is mediated by third parties – the parents’. Issues with competing values in patient-centered care were also raised in studies on adolescent and adult DSD care (Creighton & Wood, 2013; Deans et al., 2012).

Shared decision-making is recognized to have three essential elements: acknowledging that a decision is required, knowing and understanding the best available evidence, and incorporating the patient’s values and preferences into the decision (Légaré & Witteman, 2013). Studies show that shared decision-making can improve empowerment in the affected individual/parents as it helps individuals take control over their condition and lives (Fumagalli et al., 2015; Siminoff & Sandberg, 2015; Weidler et al., 2019). However, the way HCPs present information regarding uncertainty surrounding the surgery is of importance for the decision being made (Timmermans et al., 2018). Some studies highlighted the importance of shared decision-making in DSD care (e.g. Gardner & Sandberg, 2018; Siminoff & Sandberg, 2015; Weidler et al., 2019). More recently, qualitative studies explored the issue of communication between HCPs and individuals with DSD and their caregivers regarding surgical interventions, pointing out the complexities of talking to children and parents about DSD (e.g. Liao et al., 2019; Roen & Hegarty, 2018; Roen et al., 2018; Roen et al., 2023). Other studies have pointed out that caregivers do not always recognize their part in the decision-making process due to their perception of surgery being necessary (Alderson et al., 2023; Crissman et al., 2011). Seen from the HCPs’ perspectives, the literature describes challenges in decision-making processes, difficulties with communication, and concerns about whether parents grasp the complexities regarding DSD and treatment options (Roen, 2019; Roen et al., 2023; Suorsa-Johnson et al., 2022)

The discussion regarding the indications, timing, procedures, and outcome evaluations for DSD surgery is ongoing (Lee et al., 2016; Weidler et al., 2023). More research into HCPs’ perspectives on surgical practices and possible dilemmas may provide further useful insights into the shared decision-making process and, therefore, improve health outcomes for individuals with DSD (Kremen et al., 2022). This study aims to describe HCPs’ perspectives on what decision-making involves and how it is applied in DSD-related surgeries in a Norwegian setting, as well as to explore how dilemmas regarding communication with affected individuals/caregivers were perceived.

## Material and methods

We adopted a qualitative and explorative design and used FGIs to address the study’s aims (Kitzinger, 1995). FGIs may provide new knowledge through the negotiation going on between participants, and the sensitivity of the subject may be easier to discuss in a group interview where the participants are acquaintances (Kitzinger, 1994). FGIs can provide access to participant’s thoughts and perceptions on topics being studied. Also, as the creation of data takes place within the group through sharing and contesting perspectives it can foster a deeper level of exploration (Kitzinger, 1995). To ensure that research questions were of relevance to affected individuals, a reference group of three females and one male with personal experiences of living with DSD was established.

### Recruitment and participants

The healthcare of children with DSD under 18 years is organized by two regional MDTs in Norway. The MDTs are comprised by HCPs with a range of specialist backgrounds. The MDTs set up monthly in-patient-clinics where they meet individuals with DSD from 0 to 18 years and their parents. Depending on the diagnosis and the age of the child, affected individuals attend regular follow-ups every year or less often. In addition, children and young people are followed up by pediatricians in their municipalities. Individuals are generally referred to the MDTs by their general practitioner or by a specialist at the local hospital. The health care service in Norway is publicly financed. The Norwegian Ministry of Health and Care Services is responsible for the hospital service providing a uniform access to health care.

HCPs were recruited via email invitation by using purposive snowball sampling. A total of fourteen HCPs participated. Eight participants identified as females and six as males, including medical doctors with specialty in endocrinology and/or pediatrics, genetics, adolescent & adult gynecology, child – and adolescent psychiatry, pediatric urology, pediatric surgery, plastic surgery and nursing. Their age ranged from 40 to over 70 years (mean = 52.1 years), while their years of experience with DSD ranged from 1 to 30 years (mean = 9.8, median = 8). Participants had clinical experiences in this field, some did not participate in decision-making regarding DSD-surgery, but were involved in clinical follow-ups of affected individuals before and after surgery. The interview guide was designed to elicit accounts of the participants’ experiences with the decision-making process on DSD-surgery, challenges related to surgery, involvements in counselling affected individuals and caregivers, how to support the shared decision-making process and what knowledge was lacking. The participants were able to elaborate on and defend their views if challenged, and to share issues raised by the interviewer and other participants (Johnson, 2014). The FGIs were conducted on a face-to-face basis and lasted from 77 to 100 minutes. One HCP participated by phone in one of the FGIs.

### Data collection

The data were collected in three FGIs, with four to five participants in each group with various professions and geographical affiliation. The FGIs were conducted in November – December 2021. Two researchers (LM and AW) led the FGIs, accompanied by a moderator (SS and a colleague). The interviews were conducted in Norwegian, audiotaped, and transcribed verbatim by LM. Reflexive summaries were written by the interviewers and moderators and included in the analysis (Neuzil et al., 2023). Measures were made to ensure that each participant got the chance to talk, and individual opinions were encouraged.

### Data analysis

The transcripts were first independently assessed by LM and then collaboratively by the researchers to ensure the use of relevant data and to provide a rich analysis. The data analysis was drawn on Braun and Clarke’s six-stage process (Braun & Clarke, 2006) and the principles of reflexive thematic analysis (Braun & Clarke, 2019). First, the transcripts were coded (and recoded) inductively by hand, based on the participants’ own words. The impact of the group setting and the interactions that occurred influenced the co-construction of meaning (Wilkinson, 1998). This included asking the questions of ‘who's talking?’ (Hydén & Bülow, 2003, p. 308), ‘Are participants speaking on behalf of themselves and their personal perspectives, their professional group or are they tending towards explaining what practice is today?’. The group dynamic might facilitate openness and disclosure as the participants can ask follow-up questions relevant to their practice and disclose differences in practice between institutions (Wilkinson, 1998). Next, the researchers searched for categories, similarities, and divergences in the data with a focus on areas of consensus and areas of disagreement between participants. Finally, the researchers agreed on the themes that represented the findings. To illustrate each theme, the researchers provided a selection of quotations, which were slightly revised to improve the readability (Braun & Clarke, 2019). Quotes from the interviews were translated from Norwegian into English. In the results, an arbitrary number was assigned to each participant (e.g. P3), and a focus group number (e.g. I2) are reported to increase readability (e.g. I3–P1). The researchers’ relation to the topics and data diverged from close to distant, which strengthened the study’s rigor (Clarke & Braun, 2021).

### Ethical considerations

This study complied with the principles of the Declaration of Helsinki. It was approved by the Norwegian Regional Committee for Medical Research Ethics, South-Eastern Norway (approval #79444) and by the Data Protection Officer, Oslo University Hospital (approval #7000898). All participants got oral and written information about the study and signed a consent letter. To preserve anonymity each participant got a personal identification number in the transcripts. All data (audio files, field notes, and transcripts), were confidential and securely stored. To ensure rigour of the study the Consolidated Criteria for Reporting Qualitative Studies (COREQ) was followed (Tong et al., 2007), the checklist is available as Supplementary Material (Appendix A).

## Results

The participants described various dilemmas affecting decisions concerning DSD-related surgeries. The dilemmas were categorized into two main themes: (a) navigating the child’s and caregivers’ needs, and (b) dilemmas due to a lack of evidence-based practice. Table 1 specifies all the dilemmas in the themes.

**Table 1.** Dilemmas.

Theme	Dilemmas	Discussion points	
**1. Navigating the child’s and caregivers’ needs**	Who should be the decision maker?	Caregivers making decisions due to expectations of normalcy on the behalf of the child.	The child’s right to make decisions about his/her body after reaching maturity.
	Maneuver caregivers expectations	HCP argues for deferral. HCP describe parents who do not push for surgery.	HCP describe parents who express a wish for surgery in order to avoid bullying, reduce stigma, and being able to wear tight fitting clothes (gym suit, swimming suit).
	Ways to reduce family stress	Good support for families in form of psychological support and follow-up, medical guidance during surgical decision-making.	To ‘normalize’ the child’s bodily ambiguity.
	The power of information	Informing of previous practice (more liberal attitudes towards surgery) resulting in parents opting for surgery.	Informing on the practice today, and not informing about a ‘solution’ that they cannot have.
	Lack of understanding of complex medical conditions	Patients and caregivers should be the primary decision makers.	Are patients and caregivers capable of understanding all the necessary information regarding the condition, surgical interventions, and debate regarding surgery?
	Lack of willingness to make the decision	Patients and caregivers should be the primary decision makers.	Patients and caregivers prefer HCP to make the decision.
	Is there a difference between male genital surgery vs female genital surgery when it comes to degree of controversy?	Proximal hypospadias surgery perceived as less controversial by HCP. Experiences with adolescents not problematizing hypospadias surgery/reporting contentedness.	Female genital reconstructive surgery perceived as controversial by HCP. Lack of follow up after 16 years may conceal negative experiences from adult perspective.
	When affected individuals express opinions conflicting with consensus, but the follow up ends when the child reaches 16 years of age	Listening to experiences expressed by affected individuals.	Following consensus?
**2. Dilemmas due to a lack of evidence-based practice**	Decision making when alternative decision paths exists	Evidence for surgery affected by rarity or low-prevalence complex diseases in removal of gonadal tissues.	Evidence against surgery affected by rarity or low-prevalence complex diseases in removal of gonadal tissues.
	Which voices are listened too?	When satisfied individuals don’t speak up.	When dissatisfied individuals speak up.
	Issues with disclosure, fear of stigma, and lack of understanding of DSD in general	Positive experiences of surgery may be undermined.	Negative experiences of surgery may be undermined.
	Evidence-based knowledge is needed during the decision-making process	More research needed.	Financial and human resources to conduct research is lacking.
	To disseminate conflicting information regarding gonadectomies in some conditions	Gonads may contribute to beneficial hormone production. Positive to giving patients and caregivers time to process the provided information as part of the shared decision-making process.	Increased cancer risk the longer you wait with gonadal removal.

As an introductory note, the dialogues in the FGIs were characterized by changing dynamics and forms. Participants could take turns in the conversation, with comments on others’ views and phrases, or elaboration on shared perspectives and construction of meaning with the other participants. The dialogue could also be focused on differences in practice, and a discussion and reflection regarding these differences, or also dwelling on shared perspectives. The participants who had a more central position in medical follow up seemed to express themselves with more confidence. HCPs with less clinical experience in DSD tended to reflect on today’s practice and expressed fewer personal opinions into the discussions. Most HCP described the dilemmas affecting children/adolescents up to age of 18, however, some also described dilemmas affecting adults (e.g. re-do hypospadias surgery or gonadectomies in adults)

### Navigating the child’s and caregivers’ needs

How to maneuver between the caregivers’ expectations of normalcy on behalf of the child and the child’s right to make decisions after reaching maturity, was considered as one of the most challenging aspects of surgical decision-making. However, this topic demonstrated a difference in practice between the MDTs and less internal consistency. Two of the FGIs had participants working in different institutions, whereas the third FGI had participants who worked in the same institution. Most of the participants involved in female genital reconstructive surgery in children reported arguing in favor of deferring surgery in their initial communications with caregivers. Yet, some HCPs had the impression that caregivers found it difficult to adopt a ‘wait-and-see’ attitude, and that caregivers opted for surgery to make genitals ‘look like most others’, in the hope that it would reduce potential negative experiences e.g. in kindergarten. This issue revealed that some HCPs described conducting female genital reconstructive surgeries in some cases, while others elaborated on how they no longer conducted this procedure due to the change in practice. One participant expressed that:

‘Regarding the [young] girls, we have stopped operating on the virilized girls. I think the last clitoroplasty we did was exactly 10 years ago. So now they grow up more or less virilized.’ (I3–P3)

This discussion continued in I3 and participants reported that they met few children with a visible enlarged clitoris.

‘I feel that few of the [young] girls we see today have a high level of [virilization]’ (I3–P1), followed up and confirmed by I3–P5: ‘I don’t think we have had a single one [with high level of virilization]. So not operating haven’t been a difficult decision.’

This topic was also discussed in I1 and I2 (both including participants from different institutions). These two FGs focused more on experiences regarding parents’ expectations.

‘We haven’t had parents pushing for [early genital reconstructive] surgery to be done either.’ (I1–P2)

‘Well … I feel that we have. We have quite a few parents who push for [surgery].’ (I1–P1)

‘Yes, my impression is that the parents are a bit pushy, they prefer things to look normal.’ (I2–P1)

Further, HCPs described a wish endorsed by the parents to prevent bullying and reduce stigma, and enable their daughters to wear tight-fitting clothes at the beach without an enlarged clitoris being visible.

‘And you have the young girls wearing bikinis, having an enlarged clitoris, and the clitoris becomes erect. Or … yes. And it is visible when they wear a gymnastics suit. […]. It isn’t always easy for them [the parents], they have good arguments for [surgery].’ (I2–P1)

‘I kind of think … Those who do this surgery. They know how complicated this surgery is, that it isn’t necessary to do more than you have to.’ (I2–P4)

The presence of good family support was highlighted as crucial in all the interviews in terms of whether the child managed to live with genitalia that deviated from what was perceived as typical for a male or female body. Psychological support and follow-up, as well as medical guidance during surgical decision-making, were emphasized as important support for family members. However, a few HCPs sympathized with the idea that one way to reduce caregivers’ stress and contribute to coping was to ‘normalize’ the child’s genitals or to remove gonads that did not accord with the assigned sex. FG discussions in I1 demonstrate the ambivalence HCP face around whether support should be parent- or child-centered:

‘It may be that we have to listen carefully to the parents’ wishes, because we know that some of them might not be able to support their child with a deformity, even if it is very insignificant.’ (I1–P5)

I1–P4 responded to this in the context of parents choosing surgery for opportunities for their children to attend kindergarten:

‘And we have met some children, where we've understood that it becomes so difficult for those parents to send that child to kindergarten, […] that they start thinking that if she isn't operated on, she can't go to kindergarten. And I think that’s wrong too’. (I1–P4)

When discussing reasons for surgery being the desired solution, one HCP reflected on whether the way information was conveyed might influence caregivers’ decisions. For instance, when the HCPs presented the history of moving to a non-surgical treatment concept, they elaborated on earlier clinical practice whereby surgical normalization was common and more accepted. The HCPs felt that caregivers who were presented with such information tended to request surgical options. This personal insight resulted in some HCPs to reflect on that they had considerable authority, meaning they had the potential to influence affected individuals’ and caregivers’ preferences, which could be perceived as an additional dilemma.

‘I’m thinking a bit about how we convey information to the parents of [young] girls with … with clitoral hypertrophy or abnormal genitalia […]. So, we are the ones who say that we used to do that [surgery], but now we no longer operate because it is agreed that the child should be allowed to decide for oneself. We are the ones informing about previous practice. So, we kind of suggest a solution, but at the same time, we say that they can’t have it.’ (I1–P4)

Others highlighted each family’s needs, and so moved the focus in the decision-making process from HCPs to parents:

‘At times, there is a huge debate about whether one should or should not [do surgery]. I don't think one can generalize it like that. It's very individual, what parents think, believe, and desire.’ (I1–P5)

Dilemmas frequently arose in the decision-making process concerning surgery because the participants argued that affected individuals and caregivers should be the primary decision makers. However, this led to HCPs questioning whether affected individuals and caregivers understood all the necessary information regarding the condition, surgical interventions, and debate regarding surgery. Additionally, one of the HCPs that followed up both children and adults, recognized that affected individuals and caregivers may be reluctant to make the necessary decisions.

‘I am trying to introduce them [affected women] to this discussion [about genital surgery]. Slightly simplified … or to invite them to investigate a bit for themselves. Take responsibility for considering this for themselves. And they just say: ‘Yeah, I’ll do what you say,’ and this happens repeatedly.’ (I3–P5)

As described, participants raised concerns about balancing the universal need for normalcy with external expectations, including those from caregivers. However, the issue of proximal hypospadias surgery seemed to be less controversial than female genital reconstructive surgery.

‘Obviously, it’s totally forbidden to operate on girls. However, for under-virilized boys, who one assumes will reach a male identity, early operations are totally accepted. Just as long as you have the parents’ consent.’ (I3–P3)

Surgeons doing hypospadias surgery experienced that adolescents did not problematize hypospadias surgery despite the complications that might arise afterwards (e.g. fistulas). In general, participants described how adolescents were content with the decision made on their behalf when they were infants. However, they discussed how the lack of follow-up after the age of 16 years could be the reason for the absence of complains regarding hypospadias surgery, as problems may occur later in life.

### Dilemmas due to a lack of evidence-based practice

A major issue in all FGIs was how best to reassure and guide affected individuals and caregivers during the decision-making process when HCPs consider evidence-based practice to be guided by contradictory literature and a lack of consensus. Experiences with current knowledge being based on small sample sizes and a lack of long-term follow-up were brought up but not questioned. Additionally, a few participants emphasized, in their experience, that the unfavorable perceptions of DSD-related surgery were based on singular anecdotal narratives. This was seen as problematic in the communication of evidence-based information and current practice.

‘It will be interesting to see, in 10 years’ time, how those who have not had [genital reconstructive] surgery experienced it. Somebody should follow up on that. Because we are aware of a lack of knowledge regarding this question. And they are not the ones who speak the loudest in the activist forums.’ (I3–P5)

Others highlighted how it might be problematic for affected adolescents or adults to voice contentedness with surgery. This was partly believed to be because HCPs anticipate that adolescents or adults were facing internalized or expected stigma. In addition, HCPs experience that there is little knowledge and understanding of DSD among colleagues and in society in general. Accordingly, both positive and negative experiences of surgery may be undermined. I1–P2 exemplified the knowledge gap through a statement supported by the rest of the participants in I1:

‘It is really important to gain more knowledge, for those of us who work with DSD and for outsiders. Even in the department where I work, people don’t know the difference between DSD and transgender. They have no idea […]. And I find it very, very unsettling that people know so little about it.’

One of the participants not performing surgery focused on their role in bridging the knowledge gap:

‘Actually, it is the regional multidisciplinary team that is responsible for the competence enhancement and disseminating information, so it really falls back on us. We still have a job to do’ (I1–P4).

In I2 and I3, more emphasis was put on a lack of financial and human resources, as well as an absence of patient registries, as major hurdles with regard to conducting longitudinal and retrospective research on the DSD population. Research is necessary to produce the evidence-based knowledge needed during the decision-making process. However, the qualitative approach is still an emerging area of research and may or may not be related to evidence of DSD-related surgery in individuals with DSD.

‘For instance, those gonadectomies, […] we have too little knowledge about what actually happens to the gonads in several of these DSD conditions. We need to do something about this lack of knowledge at some point.’ (I2–P2)

The discussions in all FGIs emphasized giving affected individuals and caregivers time to process the information provided, as part of the shared decision-making process.

‘Then there’s this thing with Swyer [syndrome], we’ve had several cases of cancer. We need time to provide them [affected individuals] with this information [about the risks and benefits of gonadectomies], so that they can understand this … For them to understand the risk and to be part of the decision-making process regarding gonadal removal.’ (I3–P5)

However, some participants recognized that more experienced and competent colleagues could communicate a greater level of confidence in their evaluation of surgery by drawing on a broader clinical picture in the decision-making process.

‘We don’t have a registry, nor data with long-term results. A lot of the information we provide and the follow-up care are built on clinical experience. So, if we are going to do this in a proper way, we need to conduct a type of follow-up procedure that we can extract results from. […]. From a short- and long-term perspective.’ (I3–P5)

## Discussion

This FGI study explored the complex dilemmas faced by HCPs who were familiar with DSD and involved in surgical decision-making in hospital care in Norway. Most notably, shared decision-making was still, almost two decades after the consensus statement, found to be influenced by fear of stigmatization and lack of evidence-based practice. HCPs described major dilemmas they faced during the decision-making process and when guiding affected individuals and caregivers, for example, how to best support and communicate with caregivers, and how to address uncertainties related to surgeries. Results show HCPs attempts to balance the interplay between concepts of normality, personal experiences and external expectations. These results shed light on the intricate considerations and challenges that HCPs encounter when guiding affected individuals and caregivers through surgical decision-making processes in the context of DSD, and are consistent with previous qualitative research in this area (e.g. Hegarty et al., 2021; Liao et al., 2019; Roen et al., 2018; Roen & Hegarty, 2018; Timmermans et al., 2018; Weidler et al., 2023).

In the last two decades, health care of children and adolescents with DSD has shifted from a focus on ‘normalizing’ surgery as the solution to the child’s difference towards a family-centered health care, where the psychosocial and family-educational needs receive more attention (Suorsa-Johnson et al., 2022). Consistent with past literature, our findings underpin that HCPs still face dilemmas and contrasting interests when counselling individuals born with DSD and their families (Chan et al., 2020; Roen et al., 2023). An example of contrasting interests is when surgical options are explained, and caregivers of infants and young children tend to perceive DSD-related surgery as a ‘way out’ of having a child that challenges ‘normality’ and a way of avoiding the feared risks of stigmatization and bullying. Similarly, prior studies have described the dialogue concerning genital surgeries between doctors and caregivers of children with DSD, confirming that surgeries were often chosen by caregivers to reduce the uncertainty regarding their child’s future (Ellens et al., 2017; Timmermans et al., 2018).

When ambivalent and opposing messages are delivered by clinicians (describing for example benefits and potential harm of surgical options), as illustrated in this study, research indicates that caregivers may still selectively follow arguments promoting surgery (Timmermans et al., 2018). Furthermore, when HCPs struggle to reconcile the prevalent belief that surgery is necessary, despite information about the potential negative consequences, and the focus on the child’s right to decide, arguments for deferral can be perceived as unclear by caregivers (Liao et al., 2019; Timmermans et al., 2018). Therefore, information that is perceived as conflicting can complicate informed consent from caregivers and affected individuals. Moreover, reflections are necessary regarding whether HCPs’ existing knowledge and treatment recommendations are based on individual experiences, attitudes, or evidence-based knowledge. Unfortunately, literature on educational interventions is scarce in DSD (Roen et al., 2023). However, the use of checklists to avoid conflicting information from various providers has proved to be useful (Graziano & Fallat, 2016) and may be a valuable tool to address sensitive issues with affected individuals and their parents. Examples of such tools are provided by e.g. Pediatric Surgeons of Phoenix ‘DSD diagnoses checklists’ (Pediatric Surgeons of Phenix, 2023). Further, the development of decision-making support tools for HCPs working with DSD show promise and should be implemented in MDTs (Karkazis et al., 2010; Lightfoot et al., 2023; Sandberg et al., 2019; Suorsa-Johnson et al., 2023).

Some HCPs found it challenging to navigate between the child’s and caregivers’ needs during the decision-making process, with caregivers being perceived to be anxious about postponing or not pursuing genital reconstructive surgery for their child. The literature supports the notion that surgeries to correct genital ambiguity are, in some cases, performed to alleviate stigma and distress in both caregivers and affected individuals (Bougnères et al., 2017). Furthermore, Kremen et al.’s (2022) recent study of caregivers’ decisions to pursue feminizing genital procedures demonstrates that pre-surgical levels of anxiety were lower in the mothers of children who did not undergo clitoroplasty and vaginoplasty than in the mothers of children who underwent such surgery, irrespective of the diagnostic severity. However, a quantitative study of caregivers of children with moderate to severe genital atypia, suggests that the caregivers’ anxiety and depressive symptoms decreased over time regardless of their children received or declined surgery, whereas illness uncertainty was predictive of caregiver distress (Roberts et al., 2020). These results indicate that HCPs should focus on affected individuals’ and caregivers’ reasoning and feelings about decisions regarding surgery, in order to help them explore their own motivations for choosing or declining surgery. Research has shown that the anxiety experienced by parents (fear of negative outcomes for the child) is not made explicit in many clinical contexts, leaving parents with little guidance in how to deal with their fears (Suorsa-Johnson et al., 2023). Given that strong emotions affect individuals’ ability to process information that is given, addressing parents’ anxiety in clinical contexts may strengthen the decision-making process. Yet, the dilemma of the child’s right to decide regarding their body (principle of autonomy) and the dilemma involved in whether the decision maker has fully understood the implications of surgery (principle of informed consent) need to be considered. When the knowledge base is limited regarding the consequences of surgeries, as experienced by our participants, physicians need to question whether the foundations for informed consent are in place. Further, the experiences collected in this study hint at a testimonial injustice occurring between HCPs and affected individuals with DSD. Testimonial injustice is when an individual’s testimony is given less credibility because he or she belongs to a marginalized group, for example individuals with mental illnesses (Carel & Kidd, 2014). This form of discrimination may be particularly relevant for individuals fear of being stigmatized related to their DSD condition as they describe their lived experiences or treatment preferences to HCPs. Prejudice may hinder HCPs (the listener) from giving justice, meaning, or credibility to the affected individual (the speaker’s voice) due to interpreting its meaning in accordance with their prejudice (Fricker, 2017). Another example is that HCPs may use language that consists of mostly medical terminology that places their knowledge in a higher position than their patients, in this case, affected individuals risk only talking to HCPs about their DSD condition (Roen, 2019).

DSD-related hypospadias surgeries were considered less problematic than female genital reconstructive surgery by most HCPs in this study. The lack of feedback concerning postsurgical regret from affected individuals and giving boys the chance to grow up with a ‘normal’ looking and functioning penis were highlighted as reasons to perform surgery. There might be several reasons for the apparent difference in perspectives regarding male and female genital surgery. First, little surgical regret has been reported in relation to male reconstructive surgery by caregivers (Ellens et al., 2017) or affected individuals (Flewelling et al., 2022). Furthermore, the gender-wise differential effect of shame and cultural assumptions were suggested as reasons for viewing ‘normalizing’ surgery as an alternative for boys with hypospadias while problematizing female genital reconstructive surgery. Second, the World Health Organization has called for a moratorium on female ‘normalizing’ surgery, although it has not done the same regarding male ‘normalizing’ surgery (Earp et al., 2021). Relatedly, a new Icelandic law states that no permanent changes to sex characteristics, other than hypospadias surgery, can be made while the child is too young to provide consent (Alaattinoglu, 2022), which is in consistency with other countries’ laws (e.g. Malta and Germany) (Danon et al., 2023). A moratorium on surgery is also supported by human-rights activists (InterACT & Watch, 2017). While the bioethical literature has discussed the contradistinction between female and male genital surgeries, the medical and psychological literature has focused less on this issue, rendering it less applicable for HCPs (Earp et al., 2021). Roen and Hegarty (2018) found in a qualitative study that the way information was conveyed was guiding parents’ decisions towards or away from the surgery, and that by giving parents a chance to talk with a psychologist, they were empowering parents to say ‘no’ to hypospadias-surgery.

For the optimization of shared decision-making in clinical perspectives, a well-established knowledge base is required and an understanding of HCPs’ dilemmas is warranted. A lack of evidence-based knowledge may increase HCPs’ individual preferences and institutional differences (Légaré & Witteman, 2013). Evidence-based medicine is mostly based on quantitative research, and has a scientific authority (Michaels, 2021). However, we also need to amplify the voices of lived experiences of affected individuals and their families Qualitative research can contribute to bridging different viewpoints by investigating human experiences with DSD and gaining more understanding and firsthand perspectives (Carel & Kidd, 2014).

This indicates a need for shared decision-making tools that have been shown to increase affected individuals’ knowledge and improve shared decision-making (Siminoff & Sandberg, 2015). Nevertheless, prior studies have identified several obstacles relevant to the shared decision-making process concerning DSD, including cultural factors (e.g. stigma), when the affected individual’s perspective is important but difficult to obtain due to age, and when the decision makers’ choices diverge from the best available medical evidence (Légaré & Witteman, 2013). Further Roen et al. (2023) pointed out that parents should not be solely responsible for disclosing information about the condition and current or potential treatment towards their children with DSD, but that HCPs should take a more active role in this disclosure-process. Even though decision-making dilemmas are still ongoing in DSD-clinics two decades after the first consensus statement was announced (Lee et al., 2006), it became apparent in the FGIs that HCPs need to acknowledge that issues such as stigma, culture differences and the child’s age are still relevant when counselling individuals with DSD and their families. A decade after the first consensus statement, a report was published to look at changing practices and found modest improvement in some areas, but that genital surgery in infancy remains common (Michala et al., 2014).

Importantly, in a FG study it is always important to reflect on the impact of the group setting, and how the interactions that occurred influenced the co-construction of meanings and discussions (Kitzinger, 1995). It might be that the topic being presented for discussion; genital surgery in minors, is so sensitive that it was difficult for participants to present statements or engage in discussions that could be potential conflicting. As mentioned above, the participants who had a more central position in medical follow up seemed to express themselves with more confidence and were given more respect in the FGIs. Given these observations, this could be further explored in individual interviews with HPCs.

This study had several limitations. First, the results only reflect the perspectives of HCPs. It would have been useful to compare HCPs’ perspectives with affected individuals’ views on the same topics, or with the views of caregivers who have participated in the proxy-surgical decision-making process. The study participants represents a small group of HCPs, which might limit the transferability of the findings. However, the explorative and qualitative nature of the study gave rich and nuanced data that seemed to represent universal experiences among HCPs. As such, the findings may have utility across contexts (Clarke & Braun, 2021, p. 143). Another limitation is the heterogeneity of the focus groups. Homogeneity within groups is recommended to increase the ability to share experiences (Kitzinger, 1995); however, a diversity in professional backgrounds and places of employment increases the visibility of different perspectives and practices and enhances trustworthiness (Shenton, 2004). The interviewer's familiarity with medical terminology and practices might inadvertently assume a shared level of understanding, potentially leading to misinterpretation of participants’ perspectives. However, both the facilitators did not hold an insider perspective and were in a position to interrupt the conversations. Finally, only six of the HCPs conducted surgery, which might have affected some participants’ sense of the everyday relevance of the subject under study. Yet, most participants had encountered affected individuals where surgical decisions were or had been an issue. Additionally, the discussion between those who conducted surgery, and those who did not, did not reveal particular differences, but rather contributed to the explorative nature of the discussion.

## Conclusion

This study confirms the intricate dilemmas faced by healthcare professionals involved in DSD-related surgical decisions demonstrated in previous studies. The issues related to navigating caregivers’ needs, grappling with a lack of evidence-based practice, and balancing external expectations underscore the complex landscape of decision-making in this field. Addressing these challenges requires a multidisciplinary approach that encompasses comprehensive patient education, robust research efforts, and collaborative discussions among medical professionals, affected individuals, and advocacy groups. As the medical community continues to learn and evolve in its understanding of DSD, ethical and patient-centered decision-making should remain at the forefront of clinical practice. This necessitates research on the long-term consequences of undergoing or postponing surgery for those affected. Further, it is important to acknowledge that the experienced dilemmas HCPs are facing are still relevant in today’s clinical practice, and HCPs therefore need to recognize their part in the decision-making process and which voices are listened to.

Increasing knowledge in society on gender diversity in general, and DSD in particular, can help affected individuals and their families to accept their own diversity. However, they also need psychological support, and HCPs who share complete, honest, and unbiased information with affected individuals and families while using shared decision-making tools.

## Supplementary Material

Supplemental Material

## Data Availability

Given the qualitative nature of this study, the generated datasets are not publicly available due to participant confidentiality issues.
