# Supplementary material for: Differences of sex development and surgical decisions: focus group interviews with health care professionals in Norway
Source: Health Psychol Behav Med. 2024 Jul 6;12(1):2371134. doi: 10.1080/21642850.2024.2371134 (PMC11229732; doi:10.1080/21642850.2024.2371134)
Supplement: Supplemental Material [file RHPB_A_2371134_SM4328.docx]

**Appendix A**

Consolidated criteria for reporting qualitative studies (COREQ): 32-item checklist

| **No** | **Item** | **Guide questions/description** | **Response** |
| --- | --- | --- | --- |
| **Domain 1: Research team and reflexivity** |  |  |  |
| Personal Characteristics |  |  |  |
| 1. | Interviewer/facilitator | Which author/s conducted the interview or focus group? | Line Mediå conducted two FGI together with Johanna Kling. Anne Wæhre conducted one FGI together with Solrun Sigurdardottir |
| 2. | Credentials | What were the researcher's credentials? *E.g. PhD, MD* | PhD-student/registered nurse, PhD/neuropsychologist, PhD/ associate professor, PhD/MD |
| 3. | Occupation | What was their occupation at the time of the study? | PhD-student, Researcher/neuropsychologist, researcher/associated professor, researcher/research coordinator at the center for rare disorders, head of the National Treatment Service for gender incongruent children and young people at Oslo University Hospital/post doc |
| 4. | Gender | Was the researcher male or female? | All researchers were females |
| 5. | Experience and training | What experience or training did the researcher have? | Qualitative training in the master of science education program (LM). SS, LF, KBF, and AW are all experienced researchers in qualitative research and in gender incongruence. SS, LF, KBF, and AW are all experienced counsellors for several master- and PhD-students. |
| Relationship with participants |  |  |  |
| 6. | Relationship established | Was a relationship established prior to study commencement? | The interviewers (LM and AW) had met most participants prior to the interviews in network meetings, and a few in clinical settings |
| 7. | Participant knowledge of the interviewer | What did the participants know about the researcher? e*.g. personal goals, reasons for doing the research* | The participants were told about the facilitator’s professional background, and the reason for conducting the study. |
| 8. | Interviewer characteristics | What characteristics were reported about the interviewer/facilitator? e.g. *Bias, assumptions, reasons and interests in the research topic* | The facilitator’s professional background, experiences with the population, and professional experience with children, AYA and adults with DSD were reported or was already known. |
| **Domain 2: study design** |  |  |  |
| Theoretical framework |  |  |  |
| 9. | Methodological orientation and Theory | What methodological orientation was stated to underpin the study? *e.g. grounded theory, discourse analysis, ethnography, phenomenology, content analysis* | An explorative qualitative research design with an reflexive and interpretive approach underpinned the study. |
| Participant selection |  |  |  |
| 10. | Sampling | How were participants selected? *e.g. purposive, convenience, consecutive, snowball* | Participants were selected by convenience sampling |
| 11. | Method of approach | How were participants approached? e*.g. face-to-face, telephone, mail, email* | Participants received an invitation to FGI together with an invitation to a national reference network meeting from the organizer of the meeting (none of the researchers). |
| 12. | Sample size | How many participants were in the study? | 14 participants were in the study |
| 13. | Non-participation | How many people refused to participate or dropped out? Reasons? | Nine participants did not respond to the invitation, and three initially accepted but were prohibited to participate due to illness or other obligations related to the work situation.. |
| Setting |  |  |  |
| 14. | Setting of data collection | Where was the data collected? e*.g. home, clinic, workplace* | Two FGI`s were conducted at a meeting room in a hotel, and one FGItook place in a meeting room at a hospital. One participant participated by phone in one pf the FGIs. |
| 15. | Presence of non-participants | Was anyone else present besides the participants and researchers? | No |
| 16. | Description of sample | What are the important characteristics of the sample? *e.g. demographic data, date* | The participants were all healthcare professionals working with individuals born with a condition affecting sex development. Participants had different professional background) including medical doctors with specialty in endocrinology, genetics, pediatrics, adolescent & adult gynecology, psychiatry, pediatric urology, pediatric surgery, plastic surgery and nursing. Mean age was 52.1 years. Eight participants were females and six were males. |
| Data collection |  |  |  |
| 17. | Interview guide | Were questions, prompts, guides provided by the authors? Was it pilot tested? | The interview guide was drawn on the researchers previous experiences with research on DSD, published literature in the field, and clinical experience. |
| 18. | Repeat interviews | Were repeat interviews carried out? If yes, how many? | No. |
| 19. | Audio/visual recording | Did the research use audio or visual recording to collect the data? | All interviews were audiotaped and transcribed |
| 20. | Field notes | Were field notes made during and/or after the interview or focus group? | Yes, field notes were made during and after the interviews. |
| 21. | Duration | What was the duration of the interviews or focus group? | The interviews lasted between 77 and 100 minutes. |
| 22. | Data saturation | Was data saturation discussed? | Yes |
| 23. | Transcripts returned | Were transcripts returned to participants for comment and/or correction? | No |
| **Domain 3: analysis and findings**z |  |  |  |
| Data analysis |  |  |  |
| 24. | Number of data coders | How many data coders coded the data? | Two researchers double-coded all interviews (one interviewer coded all three interviews, and three researches coded one interview each) |
| 25. | Description of the coding tree | Did authors provide a description of the coding tree? | No |
| 26. | Derivation of themes | Were themes identified in advance or derived from the data? | Themes were derived from the data |
| 27. | Software | What software, if applicable, was used to manage the data? | No software were used |
| 28. | Participant checking | Did participants provide feedback on the findings? | No, but a collaboration was established with two surgeons who are familiar with DSD-related surgery to discuss the relevance of the results and the research process. |
| Reporting |  |  |  |
| 29. | Quotations presented | Were participant quotations presented to illustrate the themes / findings? Was each quotation identified? e*.g. participant number* | Yes, participant quotations were presented to illustrate the themes. An participant ID, was assigned to each participant to ensure their confidentiality. Neither gender, age, nor professional background was linked to the quotes for the same reason. |
| 30. | Data and findings consistent | Was there consistency between the data presented and the findings? | There were consistency between the data, but also deviations within the material which gave us rich and nuanced examples suitable to illuminate the experiences from the participants’ own perspectives |
| 31. | Clarity of major themes | Were major themes clearly presented in the findings? | Yes, three major themes were presented |
| 32. | Clarity of minor themes | Is there a description of diverse cases or discussion of minor themes? | Yes, deviations within the material were presented |
